# Myeloid ABCG1 Deficiency Enhances Apoptosis and Initiates Efferocytosis in Bronchoalveolar Lavage Cells of Murine Multi-Walled Carbon Nanotube-Induced Granuloma Model

**DOI:** 10.3390/ijms23010047

**Published:** 2021-12-21

**Authors:** Eman Soliman, Sophia Bhalla, Ahmed E. M. Elhassanny, Anagha Malur, David Ogburn, Nancy Leffler, Achut G. Malur, Mary Jane Thomassen

**Affiliations:** 1 Department of Internal Medicine, Division of Pulmonary, Critical Care, and Sleep Medicine, Brody School of Medicine, East Carolina University, Greenville, NC 27834, USA; solimane@zu.edu.eg (E.S.); bhallas21@ecu.edu (S.B.); malura@ecu.edu (A.M.); davidogburn4@gmail.com (D.O.); lefflern@ecu.edu (N.L.); 2 Department of Pharmacology and Toxicology, Faculty of Pharmacy, Zagazig University, Zagazig 44519, Egypt; 3 Department of Pharmacology and Toxicology, Brody School of Medicine, East Carolina University, Greenville, NC 27834, USA; elhassannya20@vt.edu; 4 Department of Microbiology & Immunology, St. George’s University, St. George 999166, Grenada; amalur@sgu.edu

**Keywords:** carbon nanotubes, lipid transporter ABCG1, efferocytosis, apoptosis, granuloma-associated lung fibrosis

## Abstract

The use of carbon nanotubes has increased in the past few decades. Carbon nanotubes are implicated in the pathogenesis of pulmonary sarcoidosis, a chronic granulomatous inflammatory condition. We developed a murine model of chronic granulomatous inflammation using multiwall carbon nanotubes (MWCNT) to investigate mechanisms of granuloma formation. Using this model, we demonstrated that myeloid deficiency of ATP-binding cassette (ABC) cholesterol transporter (ABCG1) promotes granuloma formation and fibrosis with MWCNT instillation; however, the mechanism remains unclear. Our previous studies showed that MWCNT induced apoptosis in bronchoalveolar lavage (BAL) cells of wild-type (C57BL/6) mice. Given that continual apoptosis causes persistent severe lung inflammation, we hypothesized that ABCG1 deficiency would increase MWCNT-induced apoptosis thereby promoting granulomatous inflammation and fibrosis. To test our hypothesis, we utilized myeloid-specific ABCG1 knockout (ABCG1 KO) mice. Our results demonstrate that MWCNT instillation enhances pulmonary fibrosis in ABCG1 KO mice compared to wild-type controls. Enhanced fibrosis is indicated by increased trichrome staining and transforming growth factor-beta (TGF-β) expression in lungs, together with an increased expression of TGF-β related signaling molecules, interleukin-13 (IL-13) and Smad-3. MWCNT induced more apoptosis in BAL cells of ABCG1 KO mice. Initiation of apoptosis is most likely mediated by the extrinsic pathway since caspase 8 activity and Fas expression are significantly higher in MWCNT instilled ABCG1 KO mice compared to the wild type. In addition, TUNEL staining shows that ABCG1 KO mice instilled with MWCNT have a higher percentage of TUNEL positive BAL cells and more efferocytosis than the WT control. Furthermore, BAL cells of ABCG1 KO mice instilled with MWCNT exhibit an increase in efferocytosis markers, milk fat globule-EGF factor 8 (MFG-E8) and integrin β3. Therefore, our observations suggest that ABCG1 deficiency promotes pulmonary fibrosis by MWCNT, and this effect may be due to an increase in apoptosis and efferocytosis in BAL cells.

## 1. Introduction

Sarcoidosis is a chronic granulomatous disease that affects many organs, including the lungs. Although the etiology is unknown, environmental triggers including carbon nanotubes are implicated in sarcoidosis [1,2]. The use of carbon nanotubes has increased significantly in the past few decades, and many applications including electronic industries, construction materials [3], and drug delivery [4] are possible due to their exceptional physical and biological characteristics [5]. Carbon nanotubes have also been detected in many combustion-generated contaminants [6,7]. Importantly, the incidence of “sarcoidosis-like” granulomatous disease was reported to be high among New York City Fire Department workers who were involved in the September 2001 World Trade Center (WTC) disaster where inhalation exposure to combustion materials was intense [8,9]. Subsequent analyses revealed that carbon nanotubes of various sizes and lengths were found in WTC dust samples and within the lung tissues of affected exposed individuals [1]. Therefore, environmental/occupational exposure to carbon nanotubes may trigger sarcoidosis pathogenesis. Previous studies show that both single-walled (SWCNTs) and multi-walled (MWCNT) carbon nanotubes induced pulmonary granulomatous inflammation in animal models. The pulmonary response to SWCNTs varies according to their size and dispersion [10]. Fujita et al. found that SWCNT induces chronic pulmonary granulomatous inflammation [11]; however, Mercer and colleagues reported that the well-dispersed preparation of SWCNT does not show signs of granuloma formation [12]. To the best of our knowledge, the similarities between SWCNT-induced granulomas and sarcoidosis-associated granuloma were not previously reported. Nonetheless, studies from our laboratory demonstrated that oropharyngeal instillation of multiwalled carbon nanotubes (MWCNT) in mice can induce consistent granuloma formation which is stably maintained up to 90 days after instillation and replicates human disease at multiple biological levels with histopathological features and gene expression profiles similar to those observed in sarcoidosis patients [13,14,15].

Previous studies reported elevated levels of oxidative stress markers in serum and respiratory samples along with increased apoptotic cells within the granuloma and in the bronchoalveolar lavage (BAL) cells of sarcoidosis patients [16,17,18]. In recent studies of the murine model, we demonstrated that MWCNT instilled C57BL/6 wild-type mice also have apoptotic cells in the BAL and granulomas, and this effect is accompanied by an increase in reactive oxygen species (ROS) in BAL cells, BAL fluid, and lung tissues, suggesting the role of oxidative stress and apoptosis in granuloma formation [19].

Dysregulation of the pulmonary surfactant metabolism in alveolar macrophages is another common feature of human pulmonary sarcoidosis and the MWCNT murine model [20]. Peroxisome proliferator activator receptor (PPARγ) and Liver X receptor (LXR)-regulated lipid transporters ATP binding cassette (ABC), ABCA1 and ABCG1 facilitate cholesterol and phospholipids efflux from alveolar macrophages [21,22]. PPARγ as wells as both ABCA1 and ABCG1 are downregulated in sarcoidosis patients and MWCNT instilled mice [23,24]. Our recent studies using myeloid-specific knockout mice show that deficiency of ABCG1 but not ABCA1 increases granulomatous inflammation and promotes fibrosis by MWCNT [25].

Although sarcoidosis may resolve spontaneously, up to 20% of sarcoidosis patients have progressive disease and develop fibrosis with high morbidity and mortality [26]. The pathophysiology of fibrotic sarcoidosis is unknown. Therefore, understanding the mechanisms leading to the development of pulmonary fibrosis secondary to sarcoidosis will facilitate the evolution of novel therapeutic options and improve patient survival. Our previous studies suggest that deficiency of ABCG1 may be considered as one of the predisposing factors [25]. In addition to our observation, other studies also show that ABCG1-deficient macrophages are more susceptible to oxidative stress-induced apoptosis [27]. Given that the persistence of apoptotic cells exacerbates lung inflammation and fibrosis [28,29], we hypothesize that myeloid ABCG1 deficiency increases MWCNT-induced apoptosis, promoting granulomatous inflammation and fibrosis.

## 2. Results

### 2.1. Myeloid ABCG1 Deficiency Increases TGF-β Expression and Initiates Fibrosis in the Presence of MWCNT

**Figure 1:**
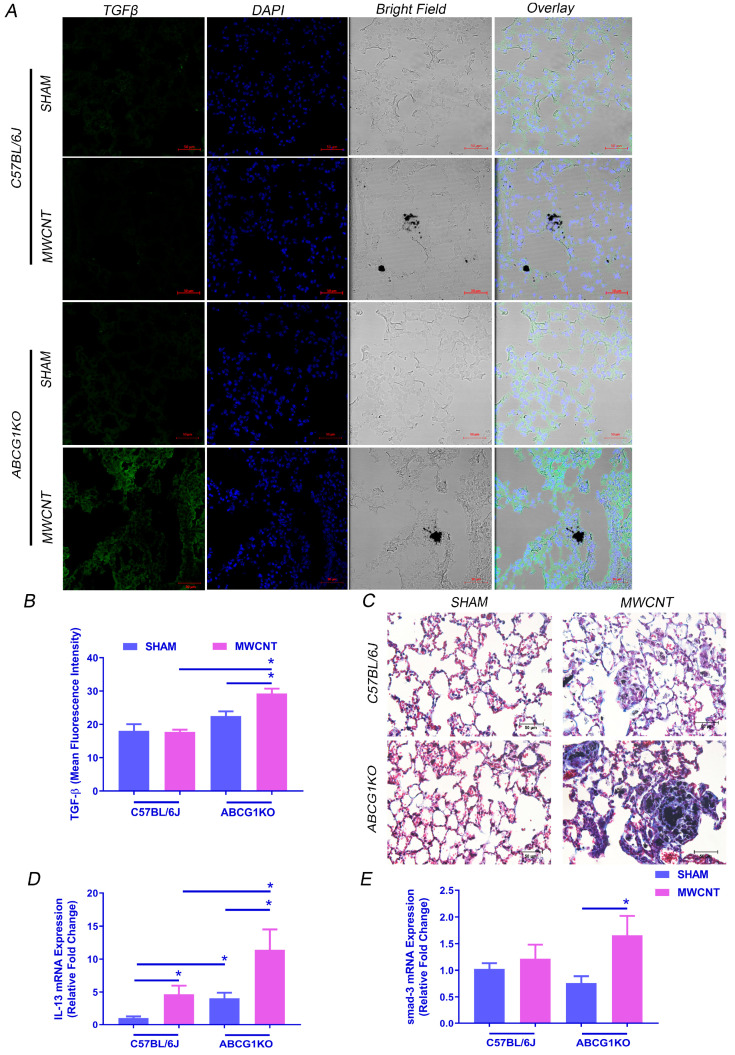
Myeloid ABCG1 deficiency increases fibrosis development and TGF-β expression in the lung of MWCNT instilled mice. (**A**) Representative images showing an increase in TGF-β expression in lung sections of MWCNT instilled ABCG1 KO mice. Lung sections from wild-type C57BL/6J mice or ABCG1 KO mice either sham- or MWCNT instilled were stained with TGF-β (green) and DAPI (blue). Bright field showing the presence of MWCNT in lung tissues. Scale bars: 50 μm. (**B**) Graphical representation of TGF-β mean fluorescence intensities using Zen 3.1 blue edition. (**C**) Representative light micrographs showing an increase in fibrosis in lung sections of MWCNT instilled ABCG1 KO mice using trichrome staining. Scale bars: 50 μm. (**D**,**E**) Measurement of IL-13 and smad-3 mRNA expression, respectively, in BAL cells using qRT-PCR (* *p* ≤ 0.05, *N* ≥ 3).

Our initial findings showed that MWCNT instillation induces pulmonary granulomatous inflammation and reduces the expression of lipid regulators PPARγ, ABCA1 and ABCG1 expression in BAL cells of wild type mice (C57BL/6J and ABCG1^F/F^) (Figure S1 and [23]). No significant difference was observed in the basal expression levels of PPARγ, ABCA1 or ABCG1 in BAL cells between C57BL/6J and ABCG1^F/F^; therefore, C57BL/6J mice were used as a wild type control. Our previous studies reported that myeloid deficiency of ABCG1 (not ABCA1) promotes granulomatous inflammation and fibrosis [25]. Consistent with these observations, MWCNT instillation induced fibrosis in myeloid-specific ABCG1 KO mice as indicated by an increase in TGF-β expression by immunofluorescence assay (Figure 1A,B and Figure S2) as well as trichrome staining of lung tissue (Figure 1C). Importantly, this increase in TGF-β was observed only in ABCG1 KO mice instilled with MWCNT, suggesting that ABCG1 deficiency enhances the transition from granulomatous inflammation to fibrosis. Previous studies show that IL-13 stimulates TGF-β expression which leads to the development of fibrosis by activation of the Smad-3 signaling pathway [30,31]. To determine whether MWCNT increases IL-13 and Smad-3 in ABCG1 KO mice, their mRNA levels were measured in BAL cells (cell differential is shown in Table 1). Figure 1D shows that the IL-13 mRNA level was significantly higher in ABCG1 KO mice (11.4-fold from sham, *p* ≤ 0.05) as compared to wild-type control mice instilled with MWCNT (4.7-fold from sham, *p* ≤ 0.05). Interestingly, Smad-3 expression increased only in MWCNT instilled ABCG1 KO mice (1.7-fold, *p* ≤ 0.05) (Figure 1E).

### 2.2. Myeloid ABCG1 Deficiency Promotes MWCNT-Induced Apoptosis in BAL Cells

**Figure 2:**
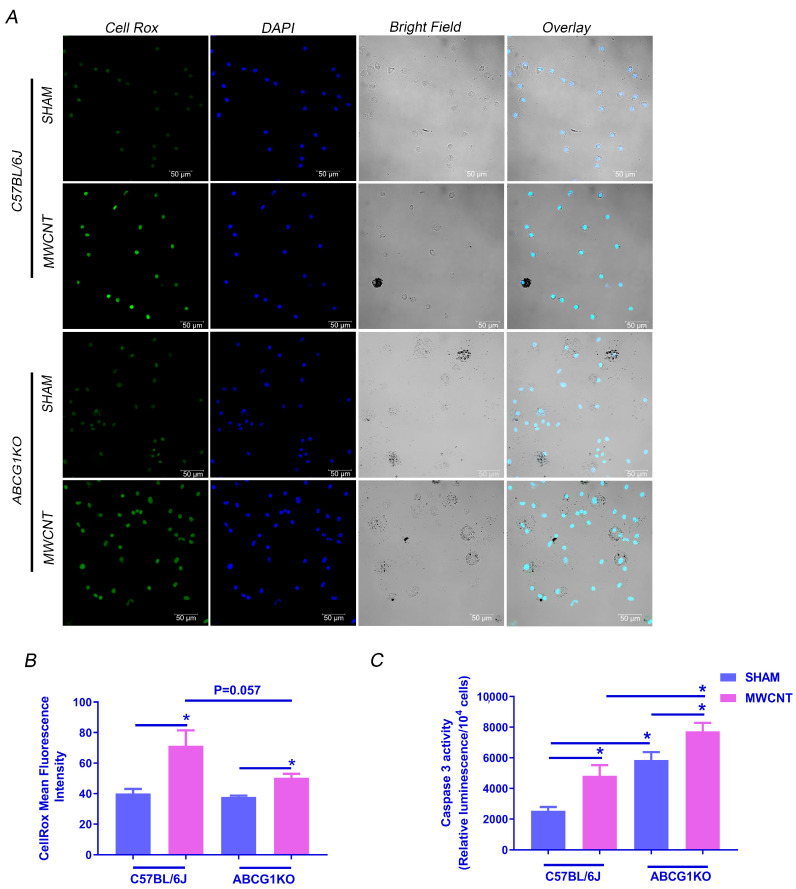
Myeloid ABCG1 deficiency promotes MWCNT-induced apoptosis in BAL cells. (**A**) MWCNT instillation increases oxidative stress in BAL cells of C57BL/6J and ABCG1 KO. Cytospins of BAL cells from C57BL/6J and ABCG1 KO mice either sham or MWCNT instilled were stained with CellRox green and nuclear stain, DAPI (blue). Scale bars: 50 μm. (**B**) Mean Fluorescence intensity was measured in 18 random fields per one cytospin for each mouse using Zen software (blue edition). (**C**) Measurement of Caspase 3/7 activity in BAL cells using Caspase-Glo 3/7 Assay and quantification of relative luminescence in 10^4^ cells. (* *p* ≤ 0.05, *N* ≥ 3).

Previous studies show that ABCG1 deficient macrophages are more susceptible to apoptosis by oxidative stress [32]. We reported that MWCNT-induced granulomatous inflammation in wild-type mice is associated with the induction of oxidative stress and apoptosis in alveolar macrophages [19]. To determine whether ABCG1 deficiency promotes MWCNT-induced apoptosis, we investigated the effect of MWCNT on oxidative stress within BAL cells of ABCG1 KO mice by live-cell fluorescence imaging using the CellRox reagent. As shown in Figure 2A,B, the fluorescence intensity was significantly elevated in MWCNT instilled wild-type and ABCG1 KO mice as compared to sham groups. Interestingly, CellRox fluorescence in MWCNT instilled ABCG1 KO mice is less than in MWCNT instilled wild-type mice (*p* = 0.057). However, apoptosis, as measured by a caspase 3/7 activity assay, was higher (1.5-fold, *p* ≤ 0.01) in BAL cells of MWCNT instilled ABCG1 KO as compared to wild-type (Figure 2C). These results suggest that ABCG1 deficient myeloid cells may be more vulnerable to MWCNT-induced apoptosis than wild-type controls, and this process is independent of oxidative stress.

### 2.3. MCWNT Activates Caspase 8-Mediated Apoptotic Pathway in ABCG1 KO Mice

**Figure 3:**
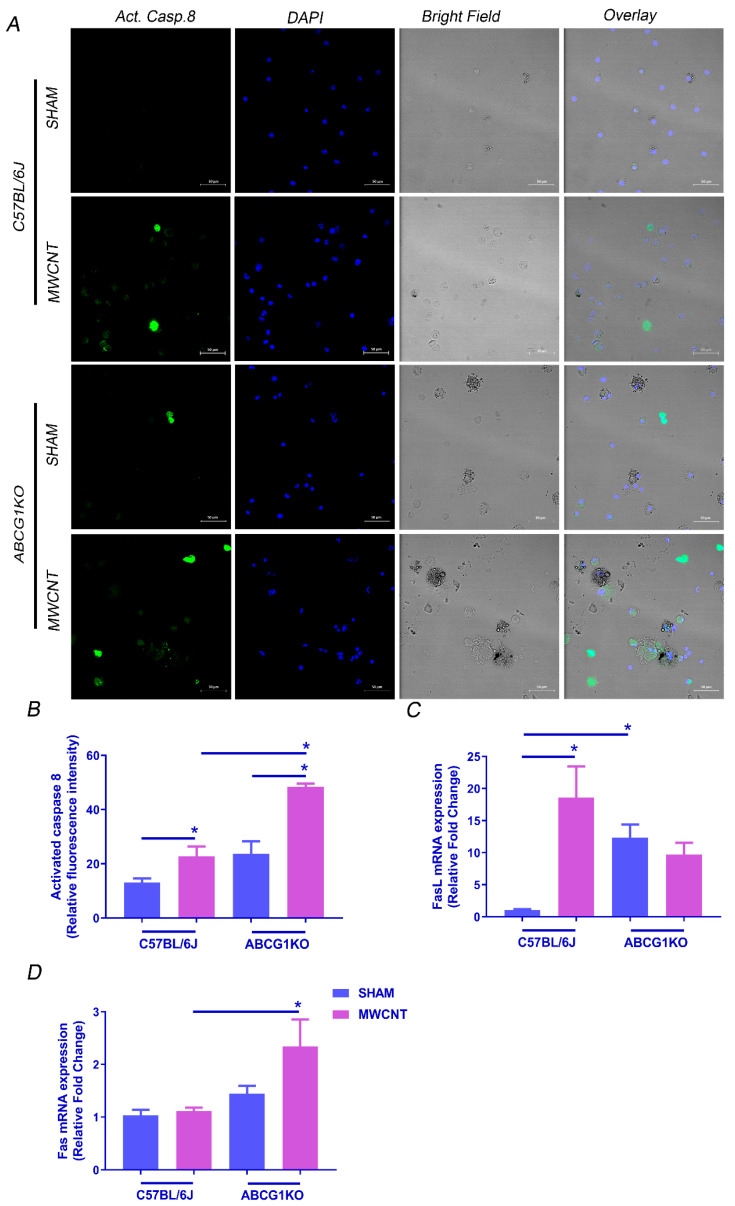
Myeloid ABCG1 deficiency increases caspase 8-mediated extrinsic apoptotic pathway by MWCNT. (**A**) Measurement of activated caspase 8 in BAL cells of wild type and ABCG1 KO mice. Cytospins of BAL cells from wild type and ABCG1 KO mice either sham or MWCNT instilled were treated with fluorescein (FITC)-conjugated IETD-FMK (green) and counterstained with DAPI (blue). Scale bars: 50 μm. (**B**) Graphical representation of activated caspase 8 mean fluorescence intensities in 18 random fields per one cytospin for each mouse using Zen 3.1 blue edition. (**C**,**D**) Measurement of mRNA expression of FasL (**C**) and Fas receptors (**D**) in BAL cells using qRT-PCR. (* *p* ≤ 0.05, *N* ≥ 3).

Apoptosis is a tightly regulated process that is activated by either extrinsic or intrinsic pathways [33,34]. Because MWCNT induced more apoptosis in BAL cells of ABCG1 KO mice than wild-type, additional studies were undertaken to ascertain which apoptotic pathway is activated and whether wild-type and ABCG1 KO mice differ. Caspase 8 is a protease enzyme that mediates the extrinsic apoptotic pathway after activation of death receptors such as Fas [35]. To determine if MWCNT induces apoptosis via the extrinsic pathway, we measured caspase 8 activity along with Fas and Fas ligand (FasL) expression. As expected, an elevation in caspase 8 activity was observed in wild-type mice (1.7-fold, *p* ≤ 0.05), and a further increase was seen in ABCG1 KO mice (2-fold, *p* ≤ 0.01) (Figure 3A,B). Moreover, the FasL mRNA level in BAL cells was higher in the presence of MWCNT in wild-type mice (18.6-fold, *p* ≤ 0.0001); however, as it was constitutively high in ABCG1 KO mice (12.3-fold, *p* ≤ 0.0001), no additional increase was observed with MWCNT instillation (Figure 3C). In contrast, Fas expression was elevated only in ABCG1 KO mice instilled with MWCNT (2.3-fold, *p* ≤ 0.05) (Figure 3D).

The intrinsic apoptotic pathway involves members of the Bcl protein family such as anti-apoptotic, Bcl-2 and pro-apoptotic, Bax [36]. Therefore, we measured Bax and Bcl-2 mRNA expression in BAL cells to investigate whether MWCNT can activate the intrinsic apoptotic pathway. The analysis revealed no elevation in the Bax/Bcl-2 ratio in ABCG1 KO mice instilled with MWCNT as compared to wild-type mice (Figure S3). These data suggest that MWCNT promotes caspase-8-mediated activation of the extrinsic apoptotic pathway and not the intrinsic pathway in BAL cells of ABCG1 KO mice.

### 2.4. MWCNT Increases Efferocytosed Apoptotic Cells in BAL of ABCG1 KO Mice

**Figure 4:**
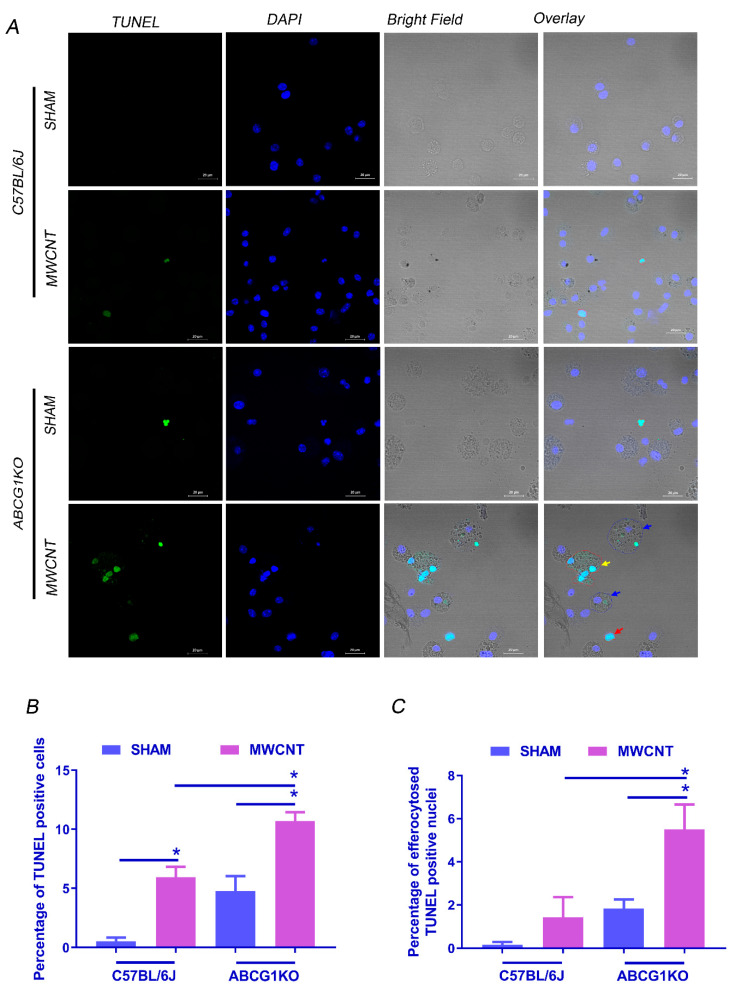
Myeloid ABCG1 deficiency increases the percentage of efferocytosed apoptotic cells after MWCNT instillation. (**A**) Representative images showing TUNEL staining for BAL cells (40×). Cytospins of BAL cells from C57BL/6J and ABCG1 KO mice either sham or MWCNT instilled were stained with TUNEL reagent for DNA fragmentation (green) and nuclear stain, DAPI (blue). Bright field showing non-engulfed apoptotic cells (red arrow), efferocytosed TUNEL+ nuclei (blue arrow) and multi-nucleated apoptotic cell (yellow arrow). (**B**) Graphical representation of the percentage of TUNEL positive cells in BAL cytospins. (**C**) Graphical representation of the percentage of efferocytosed TUNEL positive cells. Quantification of TUNEL staining was performed blindly on 18 random 20× fields per one cytospin for each mouse. *, significant difference, *p* ≤ 0.05, *N* ≥ 3.

Several studies demonstrated that the persistence of apoptotic cells in the alveolar space induces fibrosis via initiation of efferocytosis, a process which mediates the production of the profibrotic cytokine, TGF-β [37,38,39]. To determine the effect of myeloid ABCG1 deficiency on the efferocytosis process in MWCNT-induced granulomatous inflammation, the TUNEL assay was used to detect apoptotic nuclei in BAL cells, and then the number of live cells engulfing TUNEL+ nuclei was blindly counted. Consistent with our apoptosis observations, the percentage of non-engulfed apoptotic cells (cells having one or more DAPI^+^TUNEL^+^ nucleus and no DAPI^+^TUNEL^-^ nucleus) in ABCG1 KO mice with MWCNT was significantly higher than MWCNT instilled wild-type mice (*p* ≤ 0.01) (Figure 4A,B). In addition, the percentage of efferocytosed apoptotic cells was significantly higher in MWCNT-instilled ABCG1 KO mice than in the wild-type control (Figure 4C). Cells having one or more DAPI^+^TUNEL^+^ nucleus and at least one DAPI^+^TUNEL^-^ nucleus are considered efferocytes (Figure S4). Given that the engulfment of apoptotic cells initiates the production of TGF-β [40,41], our findings suggest the possible correlation between increased efferocytosis and fibrosis in MWCNT instilled ABCG1 KO mice.

### 2.5. MWCNT Increases MFG-E8-αvβ3/5 Pathway in BAL Cells of ABCG1 KO Mice

**Figure 5:**
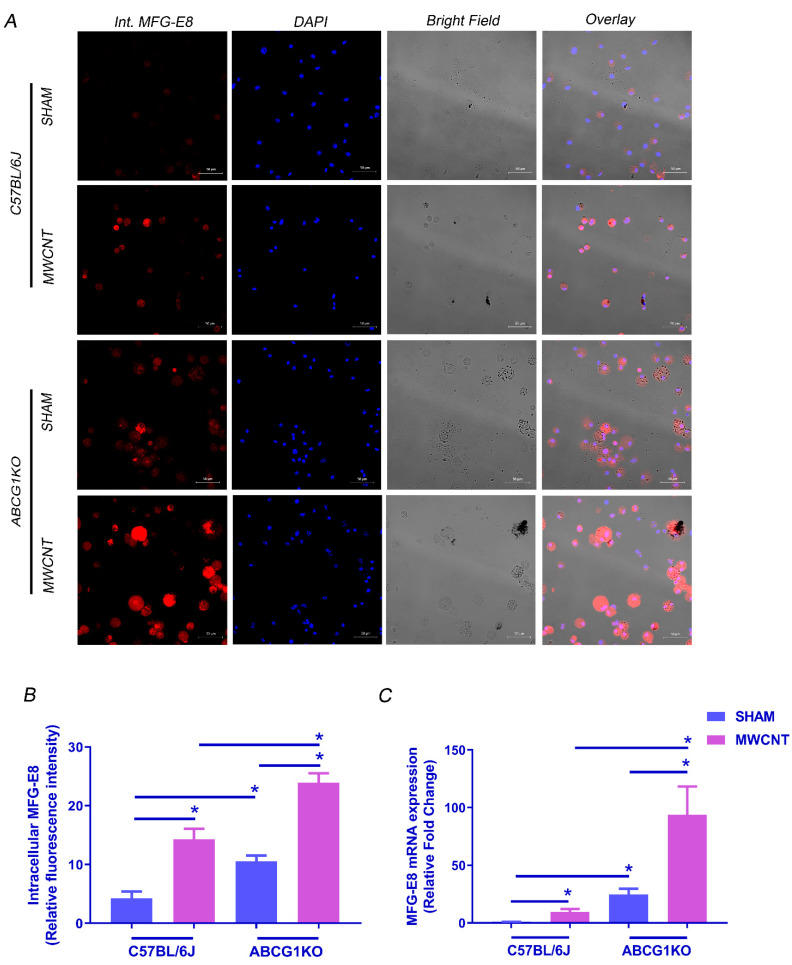
Myeloid ABCG1 deficiency increases MFG-E8 expression in BAL cells after MWCNT instillation. (**A**) Representative images for intracellular MFG-E8 expression in BAL cells from wild-type mice or ABCG1 KO mice either sham or MWCNT instilled. BAL cytospins were fixed permeabilized and stained with MFG-E8 (red) and nuclear stain, DAPI (blue). (**B**) Graphical representation of the MFG-E8 mean fluorescence intensities using Zen 3.1 blue edition. (**C**) Measurement of mRNA expression of MFG-E8 in BAL cells was measured using qRT-PCR. (* *p* ≤ 0.05, *N* ≥ 3).

**Figure 6:**
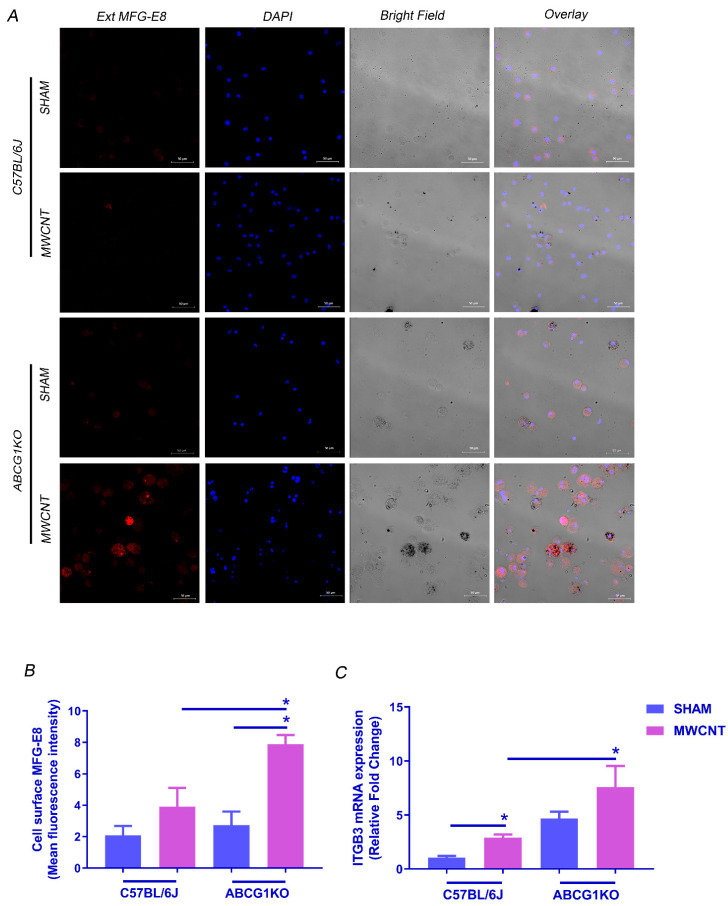
Myeloid ABCG1 deficiency increases cell surface bound MFG-E8 and ITGB3 expression in BAL cells after MWCNT instillation. (**A**) Representative images for cell surface localization of MFG-E8 in BAL cells from wild-type mice or ABCG1 KO mice either sham or MWCNT instilled. Non-permeabilized BAL cytospins were stained with MFG-E8 (red) and nuclear stain, DAPI (blue). (**B**) Graphical representation of the MFG-E8 mean fluorescence intensities measured using Zen 3.1 blue edition. (* *p* ≤ 0.05, *N* ≥ 3). (**C**) Measurement of mRNA expression of ITGB3 in BAL cells using qRT-PCR. (* *p* ≤ 0.05, *N* ≥ 3).

Efferocytosis can be mediated by MFG-E8, a bridging protein that is released from macrophages to bind phosphatidylserine (PS) on the surface of apoptotic cells. MFG-E8-opsonized cells are then recognized by integrin receptors αvβ3 or αvβ5 on macrophages for internalization [42]. To investigate whether efferocytosis in ABCG1 KO macrophages is associated with an increase in MFG-E8-αvβ3/5 signaling, levels for MFG-E8, integrin αv (ITGAV), integrin β3 (ITGB3) and integrin β5 (ITGB5) were measured. An increase in MFG-E8 mRNA and protein (Figure 5A–C and Figure S5) was observed in BAL cells of both wild-type and ABCG1 KO MWCNT instilled mice. MFG-E8 expression is constitutively high in ABCG1 KO sham mice as compared to wild-type, but a further increase was observed after MWCNT instillation as compared to MWCNT instilled wild-type mice. Since MFG-E8 is released from macrophages to bind apoptotic cells, cell surface localized MFG-E8 in non-permeabilized BAL cytospins was measured (Figure 6A,B and Figure S6). Importantly, only MWCNT instilled ABCG1 KO mice demonstrated a significant increase in cell surface MFG-E8 when compared to wild-type. This observation is consistent with an increase in efferocytosed apoptotic cells. The analysis of ITGAV and ITGB5 mRNA revealed no significant difference between MWCNT instilled wild-type and ABCG1 KO mice (Figure S5). However, a significant increase in ITGB3 level was seen in BAL cells of MWCNT instilled compared to wild-type sham instilled mice (2.9-fold, *p* ≤ 0.001). ABCG1 KO mice showed constitutively high levels of ITGB3 when compared to wild-type (4.7-fold, *p* ≤ 0.001), and MWCNT instillation resulted in a further increase in this expression (Figure 6C). These results suggest that ABCG1 deficiency may promote the MFG-E8-αvβ3/5 signaling pathway and may initiate efferocytosis in the BAL of ABCG1 KO mice after MWCNT instillation. 

## 3. Discussion

We previously demonstrated that MWCNT instillation induces pulmonary granulomatous inflammation in mice, a model that recapitulates human pulmonary sarcoidosis [15]. Dysregulation of the lipid metabolism in alveolar macrophages and downregulation of the lipid transporter ABCG1 are common features in MWCNT-induced and human pulmonary granulomatous inflammation [23]. The use of myeloid-specific ABCG1 KO mice promoted MWCNT-induced granuloma formation and fibrosis [25]. In the present study, we utilized MWCNT in ABCG1 KO mice to understand the mechanisms that cause fibrosis. MWCNT instillation increased TGF-β expression in the BAL cells [25] and the lungs of ABCG1 KO mice and elevated mRNA levels of TGF-β related signaling molecules IL-13 and Smad-3 in BAL cells. ABCG1 deficiency showed elevation in both apoptosis and efferocytosis in BAL cells of MWCNT-instilled mice when compared to wild-type. This effect was associated with activation of the MFG-E8-αvβ3/5 efferocytosis pathway. Given that persistent apoptosis aggravates lung inflammation and efferocytosis increases TGF-β production, our observations suggest that MWCNT may increase both apoptosis and efferocytosis events in BAL cells of ABCG1 KO mice, resulting in enhanced chronic granulomatous inflammation and fibrosis.

Oxidative stress caused by the over-production of reactive oxygen species (ROS) plays a pivotal role in apoptosis. Accumulated intracellular ROS alters mitochondrial membrane potentials, resulting in mitochondrial cytochrome C release, Bcl-2-dependent mitochondrial pH alteration and ultimately induction of apoptosis [43]. Using a murine model of chronic granulomatous inflammation, we demonstrated that MWCNT instillation induces oxidative stress resulting in mitochondrial dysfunction and apoptosis in BAL cells [19]. Previous studies show that ABCG1 reduces oxidative stress and protects against oxidative-stress-induced apoptosis [27,44]. Hence, we hypothesized that both oxidative stress and apoptosis would be higher in BAL cells of ABCG1 KO mice after MWCNT instillation. Surprisingly, less oxidative stress but more apoptosis was observed in ABCG1 KO mice than wild-type. In addition, BAL cells of these mice showed more caspase 8 activation and death receptor, Fas, expression. These observations suggest that myeloid ABCG1 deficiency enhances MWCNT-induced apoptosis, and this process may be dependent on the extrinsic apoptotic pathway and independent of oxidative stress.

Apoptosis is physiologically important for the development and maintenance of tissue homeostasis. Under normal physiological conditions, apoptotic cells are usually undetectable in the lung due to a balance between apoptosis and the clearance of apoptotic cells by airway macrophages via a process called efferocytosis [45]. Studies have reported the detection of apoptotic cells in BAL fluid and granulomas from sarcoidosis patients [16]. We demonstrated that MWCNT instillation increases apoptotic cells in BAL fluid and lung tissues of wild-type mice [19], and ABCG1 deficiency exacerbated apoptosis in BAL cells and promoted pulmonary fibrosis. The link between apoptosis and fibrosis in sarcoid-like granulomatous inflammation is unclear. However, several studies suggest that the presence of apoptotic cells in the alveolar space may be involved in the pathogenesis of pulmonary fibrosis [29,37,38,39]. The fibroproliferative response in the lung of bleomycin-treated mice is associated with alveolar macrophage apoptosis [46]. In addition, intratracheal administration of apoptotic macrophages increases collagen deposition and induces pulmonary fibrosis with upregulation of fibrogenic TGF-β and matrix metalloproteinases [39]. Herein, we found that MWCNT increased apoptosis in BAL cells of ABCG1 KO mice along with the increased TGF-β expression and fibrosis. These observations suggest that ABCG1 deficiency promotes apoptosis which may initiate fibrosis in sarcoid-like granulomatous inflammation.

One important mechanism for apoptosis-induced pulmonary fibrosis is mediated by eat-me and post-engulfment signaling of macrophage efferocytosis [37]. The efferocytosis process regulates tissue homeostasis and inflammation resolution by releasing the anti-inflammatory cytokines from efferocytes. One of these cytokines is TGF-β, the most well-known pro-fibrotic mediator [47]. Previous studies show that a single instillation of apoptotic Jurkat cells in bleomycin-stimulated lungs in mice resulted in initial upregulation of bleomycin-induced TGF-β followed by downregulation of its expression at the later phase. This effect was accompanied by a reduction in apoptosis, resolution of inflammation, and alleviation of fibrosis [48,49]. However, persistence of apoptosis may lead to an ongoing engulfment of apoptotic cells by macrophages resulting in pulmonary fibrosis rather than inflammation resolution [37,38]. Wang et al. reported that a single intratracheal administration of apoptotic macrophages induces chronic pulmonary fibrosis due to continuous induction of caspase 8-dependent secondary apoptosis along with ongoing efferocytosis [38,39]. In addition, Kim and colleagues demonstrated that repetitive intrapulmonary administration of apoptotic type II alveolar epithelial cells induces lung fibrosis which is mediated by the continuous efferocytosis process [37]. Consistently, our previous and current studies showed that ABCG1 KO mice instilled with MWCNT exhibit a chronic increase in TGF-β in BAL fluid [25] and lung tissues along with pronounced fibrosis with the absence of any sign of inflammation resolution. We also found a significant increase in both apoptosis and efferocytosis in BAL cells of ABCG1 KO mice 60 days post-MWCNT instillation. We speculate that the persistence of apoptotic cells in the alveolar space of MWCNT-instilled ABCG1 KO mice is likely to contribute to prolonged TGF-β expression because of continuous apoptotic cell phagocytosis therefore maintaining fibrosis rather than resolution.

Previous studies show that the PPARγ/ LXR/ ABCG1 signaling pathway is important for efficient clearance of apoptotic cells and the resolution of inflammation [50]. After ingesting apoptotic cells, intracellular content increases and membrane-derived lipids accumulate in the efferocytes. Reverse cholesterol transport machinery, including the ABCG1 transporter, is initiated after engulfment to reduce lipid accumulation and ensure the survival of macrophages post-efferocytosis [51,52]. Interestingly, macrophages lacking this transporter have a high capacity for the engulfment of apoptotic cells; however, these macrophages may undergo apoptotic death after efferocytosis [27,44]. In the present study, we observed an increase in efferocytosed apoptotic bodies in BAL cells of ABCG1 KO mice instilled with MWCNT; however, it was not possible to determine if ABCG1-deficient phagocytes undergo post-efferocytosis apoptosis resulting in the persistence of apoptosis in alveolar space. Due to the large number of multinucleated macrophages in the BAL of ABCG1 KO mice, it was difficult to distinguish between efferocytes undergoing apoptosis and apoptotic multinucleated macrophages; both have more than one DAPI^+^TUNEL^+^ nucleus with no DAPI^+^TUNEL^-^ nucleus. Accordingly, post-efferocytosis apoptosis of ABCG1-deficient macrophages in granulomatous inflammation requires further investigation.

Apoptotic cells control their own clearance by activating caspases and increasing the cell surface expression of phosphatidylserine to engage phagocytic receptors and facilitate the engulfment [41,53]. Macrophages recognize these apoptotic cells and secrete efferocytotic bridge molecules to engulf dead cells [41]. MFG-E8 is one of the bridging molecules that is expressed on the cell surface or released upon recognition of apoptotic cells. MFG-E8 along with αvβ3/5 integrin/vitronectin receptors then bind phosphatidylserine on the apoptotic cell surface to facilitate the corpse internalization [41,54]. In the present study, we observed apoptotic BAL cells in MWCNT instilled wild-type mice and MWCNT instilled ABCG1 KO mice. However, efferocytosis was observed only in ABCG1-deficient mice instilled with MWCNT. Efferocytosis in MWCNT instilled ABCG1 KO mice was accompanied by increased expression of caspases (-8 and -3/7), MFG-E8 (mRNA and protein) and integrin ITGB3 when compared to MWCNT instilled wild-type mice. These observations suggest that both apoptosis and efferocytosis occur concurrently in BAL of ABCG1 KO mice in the presence of MWCNT.

The efferocytosis bridging molecule, MFG-E8, along with αv integrin receptors regulate tissue fibrosis. Atabai et al. (2009) showed that macrophage MFG-E8 reduced the severity of pulmonary fibrosis via enhancing cellular uptake and the clearance of accumulated collagen [55]. Interestingly, this effect is independent of the apoptotic cell clearance. Therefore, the observed constitutive high expression of MFG-E8 (mRNA and intracellular protein levels) in ABCG1 KO mice (sham instilled) in our study may explain the absence of fibrosis in the lungs of these mice. However, increased cell surface expression of MFG-E8 in MWCNT instilled ABCG1 KO mice may contribute to fibrosis by activating αvβ3/5 integrin signaling during efferocytosis [56].

One limitation in our study is that we have not determined if increasing the expression of ABCG1 in alveolar macrophages may reduce apoptosis and aid in granuloma resolution. A pharmacological agent that selectively induces ABCG1 expression and an ABCG1 overexpressing mouse model are not commercially available. In addition, due to the chronic nature of MWCNT-induced granulomas, we were not able to replicate a similar inflammation milieu in vitro.

In conclusion, the current study proposes a potential role of ABCG1 in fibrosis secondary to a chronic pulmonary granulomatous inflammation induced by MWCNT. Engulfment of MWCNT by alveolar macrophages leads to a chronic increase in apoptosis and efferocytosis in the alveolar space. This efferocytosis process may result in pulmonary fibrosis with persistent granulomatous inflammation. Future studies are required to investigate the molecular mechanism of the efferocytosis process in ABCG1-deficient macrophages and to determine if enhancing ABCG1 expression may reduce apoptosis and ultimately aid in granuloma resolution.

## 4. Materials and Methods

### 4.1. Preparation of MWCNT Suspension

MWCNTs were purchased from SES Research (900–1501, lot-GS1802, Houston, TX, USA). MWCNT was suspended in 35% Infasurf solution (a gift of ONY, Inc., Amherst, NY, USA) diluted in phosphate buffered saline (PBS). The MWCNT mixture (2 mg/mL) was sonicated using an ultrasonic bath sonicator (model 1510R-MTH; Branson Ultrasonics Corp. Danbury, CT, USA) to achieve even particle distribution. A full characterization of MWCNT of lot—GS 1802 [57]. MWCNT width is approximately 20–30 nm; surface area is 85.75 m^2^/g, and the pore volume is 0.22 cm^3^/g as revealed by scanning electron microscopy. The net metal catalyst (Fe) content catalyst content is below 1 weight percent as revealed by thermogravimetric analysis.

### 4.2. Animals

Myeloid-specific ABCG1 KO mice were generated by crossing ABCG1^F/F^ with LysM^Cre+/+^ mice obtained from Jackson Laboratory [25]. C57BL/6J mice (Jackson Laboratory, Bar Harbor, ME, USA) were used as wild-type controls. Experimental procedures were approved by the institutional animal care committee at East Carolina University, Animal Use Protocol J199. Mice were housed in a controlled environment (22 ± 2 °C; 12 h light/dark cycle; food and water were provided ad libitum).

### 4.3. MWCNT Instillation

Animals were anesthetized using isoflurane inhalation followed by oropharyngeal instillation of 100μg (in 50 μL) of MWCNT or vehicle alone as the sham control. Sixty days after instillation, mice were euthanized with TBE (250 mg/Kg), and bronchoalveolar lavage (BAL) fluid was collected by aspiration of pre-warmed PBS from the lung for differential cell count and further analysis as previously described [58]. Lungs were dissected and either frozen in OCT or fixed with 10% formalin, dehydrated and paraffin embedded [13,58].

### 4.4. Gomori Trichrome Staining of the Lung

Paraffinized lung sections (5 μm) were dewaxed, rehydrated and then stained with hematoxylin (counterstain) and Gomori trichrome as previously described [25]. Images were captured using Axio Imager M2 (Zeiss, Inc., White Plains, NY, USA).

### 4.5. Immunohistochemical Analysis of TGF-β Expression in Lung

OCT frozen lung sections (5 μm) were fixed in 4% paraformaldehyde solution diluted in PBS for 15 min, washed with PBS, permeabilized with 0.2% triton X-100 in PBS and then incubated in blocking buffer (2% bovine serum albumin in 0.2% triton) for 1 h. Tissue sections were then incubated overnight (4 °C) in rabbit monoclonal anti-TGF-β (Sc-130348, Santa Cruz Biotechnology) solution in a blocking buffer (1:250), washed and then incubated with goat anti-rabbit (alexaflour 488, Invitrogen, Grand Island, NY, USA) 1:1000 solution in blocking buffer for 1 h. Slides were then washed with PBS and mounted using ProLong Antifade with DAPI (Invitrogen, Grand Island, NY, USA). Slides stained with the secondary antibody without prior incubation with the primary antibody were used as a negative staining control. Images were captured using confocal microscopy (Zeiss LSM 700, Zeiss, Inc., White Plains, NY, USA).

### 4.6. qRT-PCR Analysis

Total RNA was extracted from BAL cell pellets using the miRNeasy Micro Kit (Qiagen, Germantown, MD, USA) following the manufacturer’s protocol. Specific primers for IL-13 (Cat. No. PPM03021B), smad-3 (Cat. No. PPM04461C), FasL (Cat. No. PPM02926E), Fas (Cat. No. PPM03705B), Bax (Cat. No. PPM02917E), Bcl-2 (Cat. No. PPM02918F), MFG-E8 (Cat. No. PPM24674F), ITGAV (Cat. No. PPM03662D), ITGB3 (Cat. No. PPM03687E), ITGB5 (Cat. No. PPM03681C) and GAPDH (Cat. No. PPM02946E) were obtained from Qiagen. Contaminating genomic DNA was eliminated from RNA samples, and reverse transcription was performed to synthesize cDNA using the RT^2^ First Strand Kit (Qiagen, Germantown, MD, USA). The RT² SYBR Green qPCR Mastermix (Qiagen, Germantown, MD, USA) and StepOnePlus PCR system (Thermo Fisher Scientific, Grand Island, NY, USA) were used to perform qRT-PCR. Statistical analysis was performed on dct values. Fold change was calculated using the 2^−ΔΔCT^ method in comparison to GAPDH as described by Livak and Schmittgen [59].

### 4.7. Intracellular Oxidative Stress in BAL Cells

CellROX green (Invitrogen, Grand Island, NY, USA) was utilized for measuring oxidative stress in BAL cells. BAL cytospins were incubated with CellROX green (5 μM) for 30 min at 37 °C, fixed using 4% buffered paraformaldehyde and then mounted with ProLong Antifade with DAPI (Invitrogen, Grand Island, NY, USA). Images were obtained using confocal microscopy (Zeiss LSM 700). 

### 4.8. Measurement of Caspase 3/7 Activity

Caspase 3/7 activity was measured in 15,000 BAL cells using Caspase-Glo 3/7 reagent (Promega, Madison, WI) following the manufacturer’s instructions. Luminescence was measured using the Infinite 200 Pro plate reader (Tecan Trading AG, Männedorf, Switzerland).

### 4.9. Measurement of Caspase 8 Activity

Caspase 8 activity was measured in BAL cells using the Casp GLOW Fluorescein Active Caspase-8 Staining Kit (Invitrogen, USA) as directed by the manufacturer. BAL cytospins were incubated with the FITC-IETD-FMK reagent for 1 h at 37 °C, washed three times with the wash buffer included with the kit and then mounted with ProLong Antifade with DAPI (Invitrogen, USA). Images were captured using confocal microscopy (Zeiss LSM 700).

### 4.10. TUNEL Assay

DNA fragmentation of apoptotic BAL cells was measured using the TUNEL assay kit (In Situ Cell Death Detection Kit; Roche; Indianapolis, IN). BAL cytospins were fixed in 4% paraformaldehyde, permeabilized using 0.2% triton solution and then incubated with the TUNEL reaction mixture following the manufacturer’s instruction. Slides were then rinsed, air dried and mounted with ProLong Antifade containing DAPI (Invitrogen, USA). Images were captured using confocal microscopy (Zeiss LSM 700). The number of TUNEL+ cells was counted using image J in 18 random 20× fields in one BAL cytospin per mouse, and each group has *N* ≥ 3.

### 4.11. Immunohistochemical Analysis of MFG-E8 Expression in BAL Cells

Both intracellular and extracellular MFG-E8 expression was measured in BAL cytospins using immunohistochemical analysis. For intracellular expression, cytospins were fixed with 4% paraformaldehyde in PBS, permeabilized with 0.3% triton in PBS and blocked with 2% BSA in 0.3% triton. After blocking, cells were incubated with mouse anti-MFG-E8 (Santa Cruz Biotechnology, Dallas, TX USA) (1:150 dilution in blocking buffer) overnight at 4 °C. Cells were then washed and then incubated with goat anti-mouse (alexaflour 555, Invitrogen, Grand Island, NY, USA) (diluted 1:1000 in blocking buffer) for 1 h. For cell surface expression of MFG-E8, cytospins were fixed with 2% paraformaldehyde in PBS and blocked with 2% BSA in PBS (no triton was added) without permeabilization. After blocking, cells were incubated with mouse anti-MFG-E8 (1:150 dilution in blocking buffer without triton) at room temperature for 30 min. Cells were then washed and then incubated with goat anti-mouse alexaflour 555 (diluted 1:1000 in blocking buffer without triton) for 1 h. After incubation with the secondary antibody, cells were washed with PBS, air dried and then mounted with ProLong Antifade containing DAPI (Invitrogen, Grand Island, NY, USA). Images were captured using confocal microscopy (Zeiss LSM 700).

### 4.12. Statistical Analyses

Data are presented as the mean ± standard error of the mean (SEM). Data were analyzed using two-way ANOVA followed by the Bonferroni correction when two independent variables are involved, or a *t*-test when comparing two groups when one independent variable is involved. GraphPad Prism 7 software (GraphPad, Inc., San Diego, CA, USA) was used for statistical analysis.

## Figures and Tables

**Table 1:** Total and differential cell counts of BAL fluid of vehicle or MWCNT instilled animals.

Strain/Treatment	Total Cell Count (×10^6^)	Macrophages (%)	Lymphocytes (%)	PMN (%)
C57BL/6				
SHAM (*N* = 10)	0.8 ± 0.1	98.1 ± 0.8	1.4 ± 0.6	0.5 ± 0.3
MWCNT (*N* = 11)	0.7 ± 0.1	95.6 ± 1.1	3.1 ± 0.8	1.3 ± 0.5
ABCG1 KO				
SHAM (*N* = 16)	2.0 ± 0.2 *	90.1 ± 2.1 *	5.3 ± 0.9 *	4.7 ± 1.4 *
MWCNT (*N* = 12)	2.2 ± 0.2 *	85.1 ± 3.7 *	7.7 ± 2.0 *	7.3 ± 2.4 *

* *p* ≤ 0.05 compared to C57BL/6J same treatment. Non-significant difference was observed between sham and MWCNT-instilled mice in the same genotype.

## Data Availability

Not Applicable.

## Supplementary Materials

The following are available online at https://www.mdpi.com/article/10.3390/ijms23010047/s1.
