# Supplementary figures and images for: Myeloid ABCG1 Deficiency Enhances Apoptosis and Initiates Efferocytosis in Bronchoalveolar Lavage Cells of Murine Multi-Walled Carbon Nanotube-Induced Granuloma Model
Source: Int J Mol Sci. 2021 Dec 21;23(1):47. doi: 10.3390/ijms23010047 (PMC8744594; doi:10.3390/ijms23010047)

**A**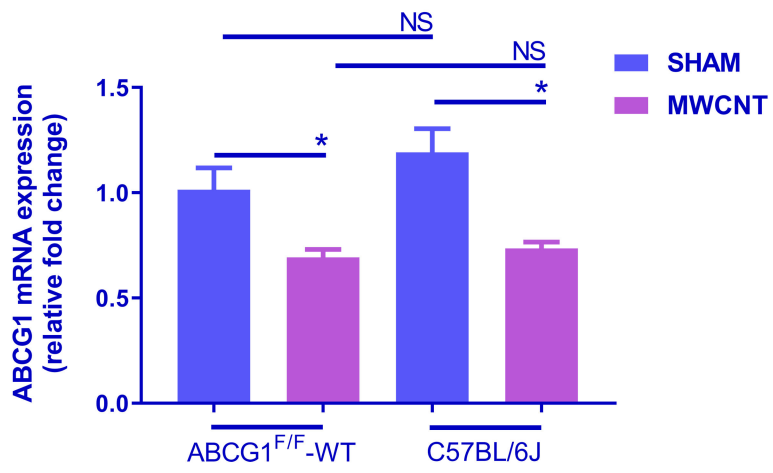**B**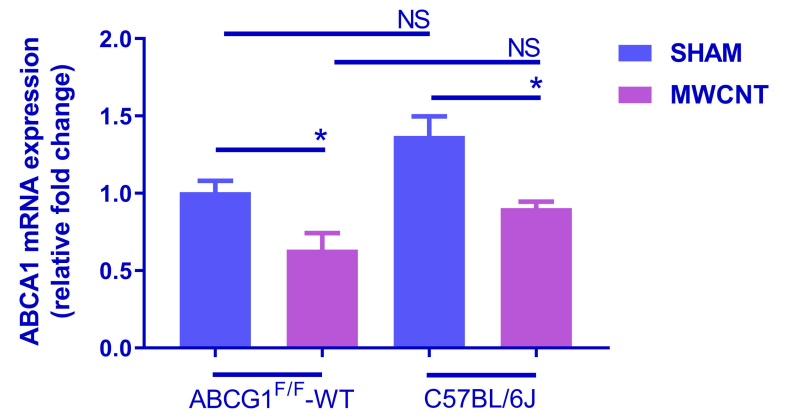**C**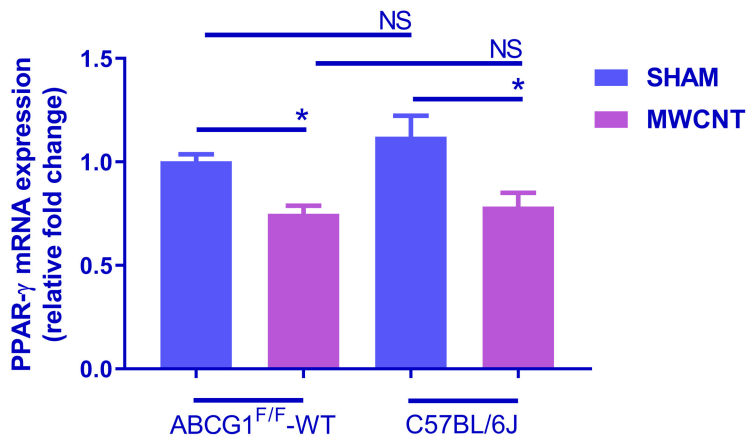

Supplement: Supplementary file 1 [file ijms-23-00047-s001.zip › supplemental figure S1.pdf]

# ABCG1 KO/ MWCNT

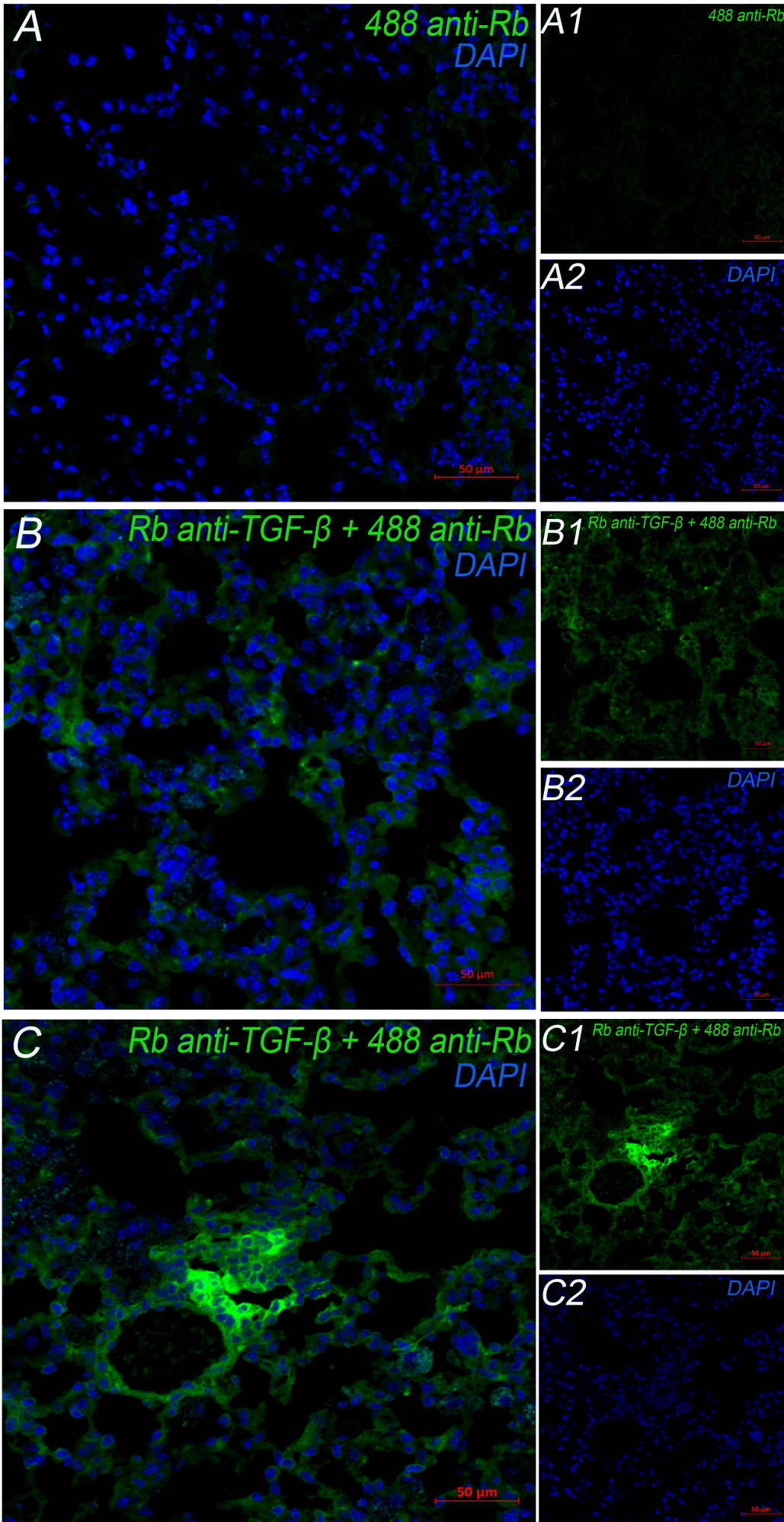

Supplement: Supplementary file 1 [file ijms-23-00047-s001.zip › supplemental figure S2.pdf]

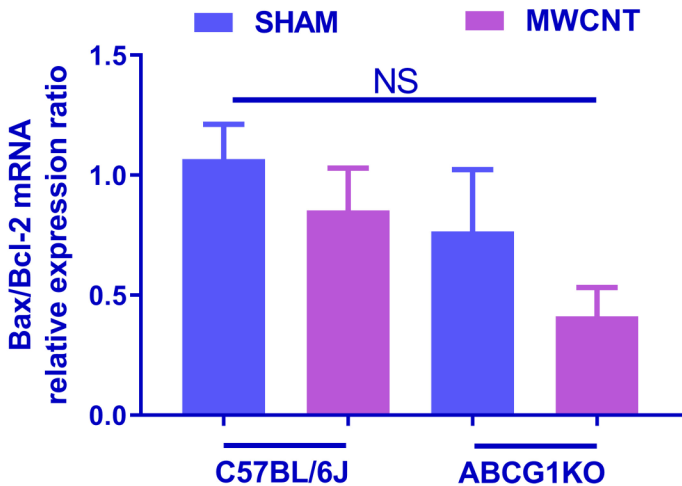

Supplement: Supplementary file 1 [file ijms-23-00047-s001.zip › supplemental figure S3-.pdf]

# ABCG1 KO/ MWCNT

Merged

DAPI/TUNEL

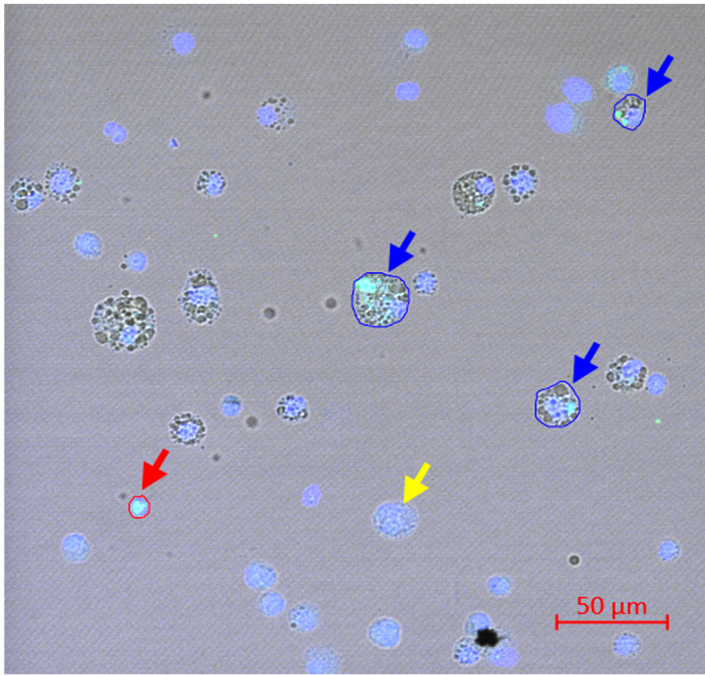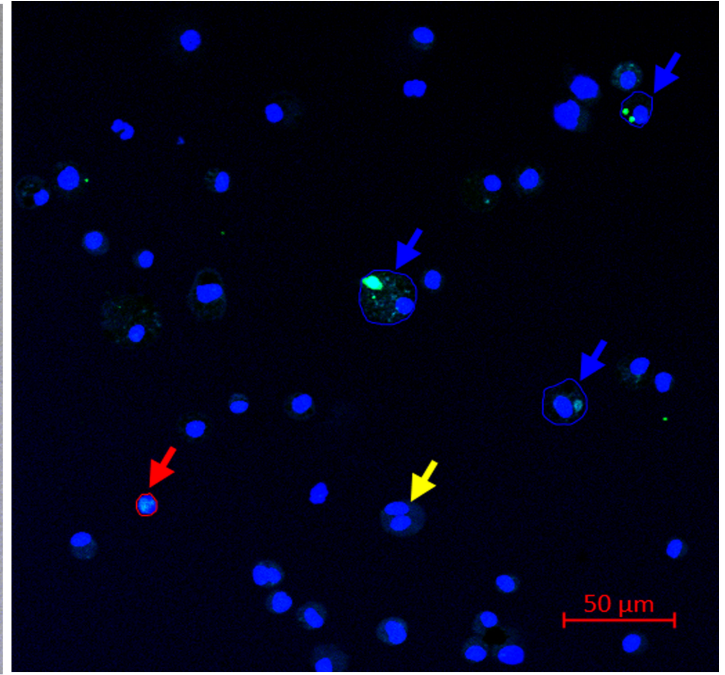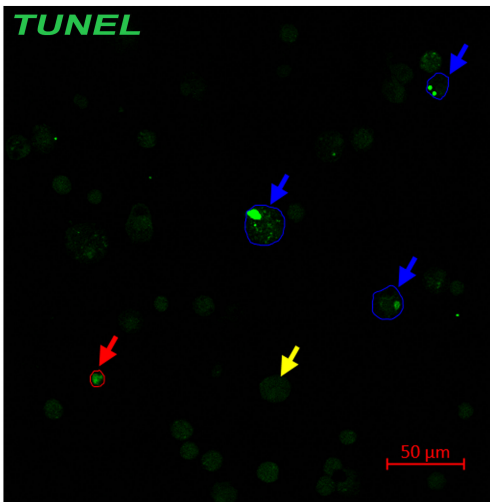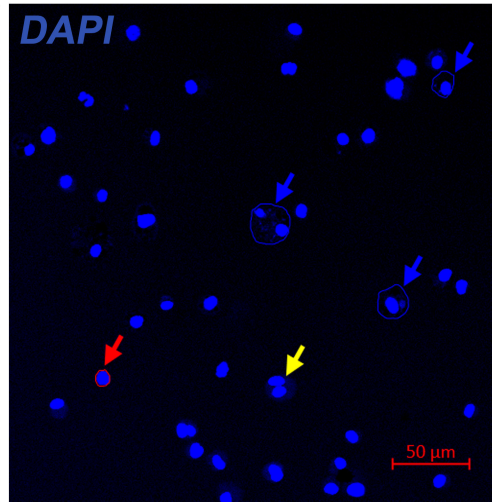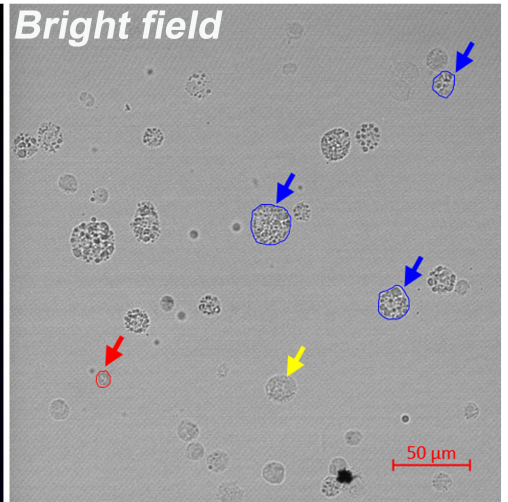

Supplement: Supplementary file 1 [file ijms-23-00047-s001.zip › supplemental figure S4.pdf]

DAPI / Int. MFG-E8

C57BL/6J

SHAM

MWCNT

ABCG1KO

SHAM

MWCNT

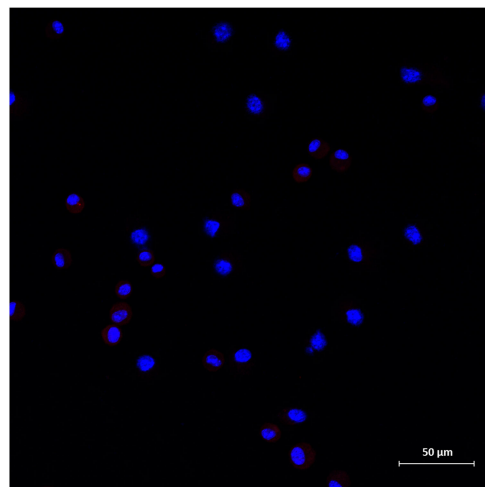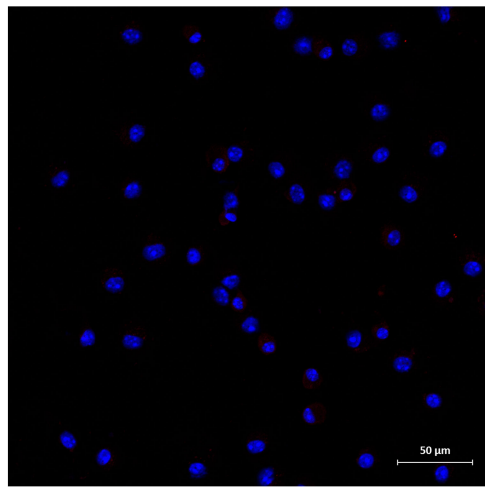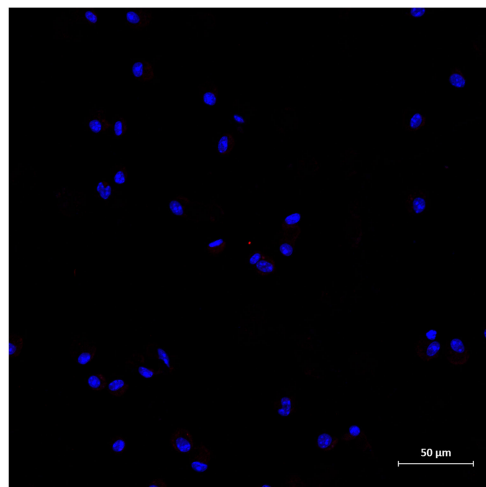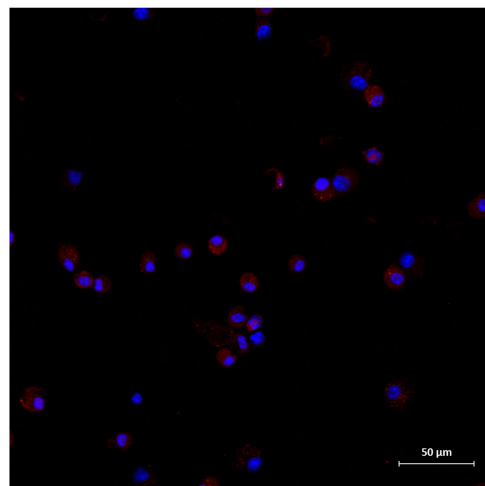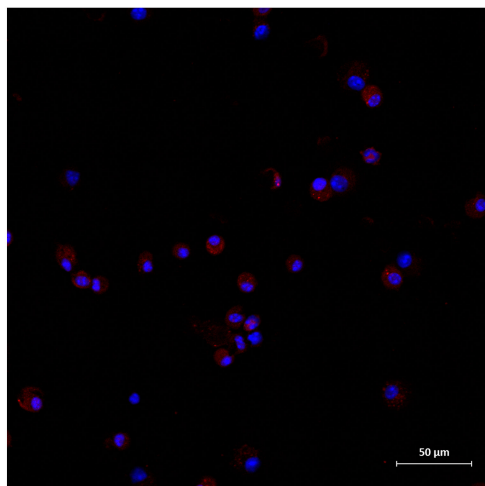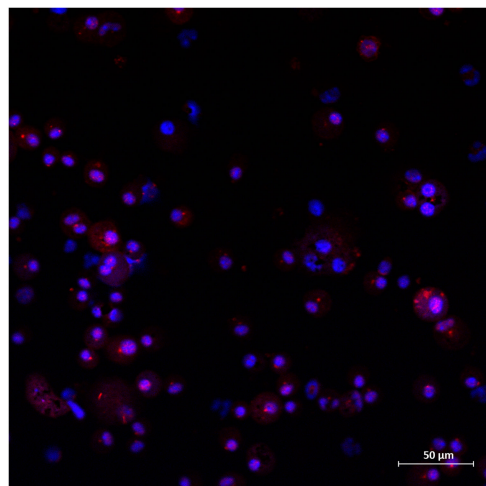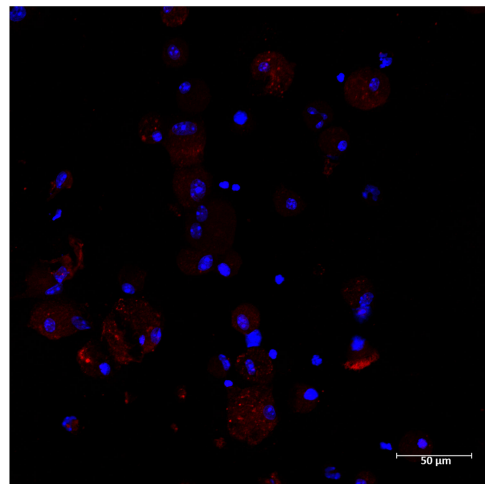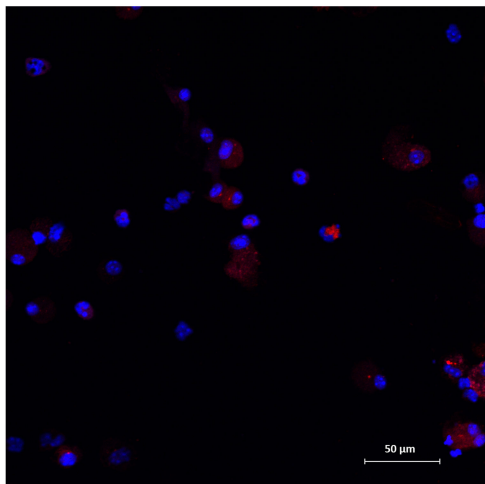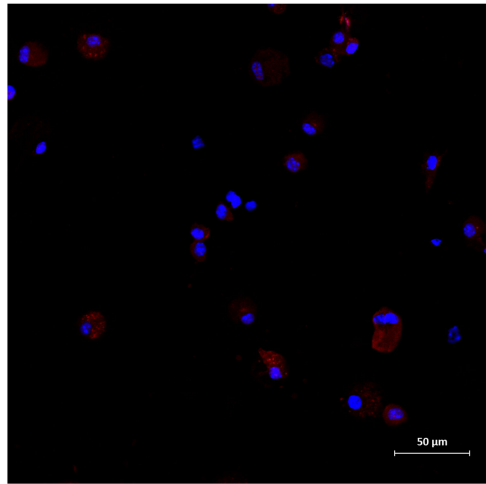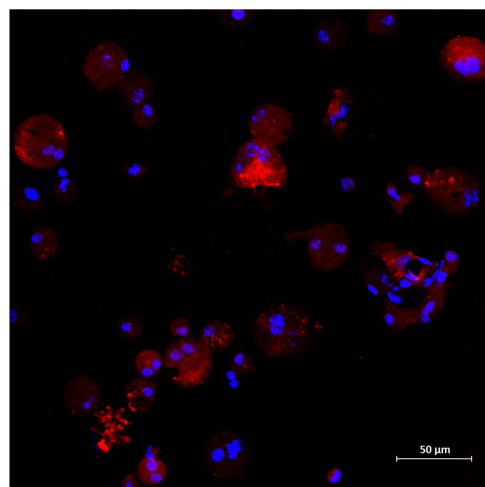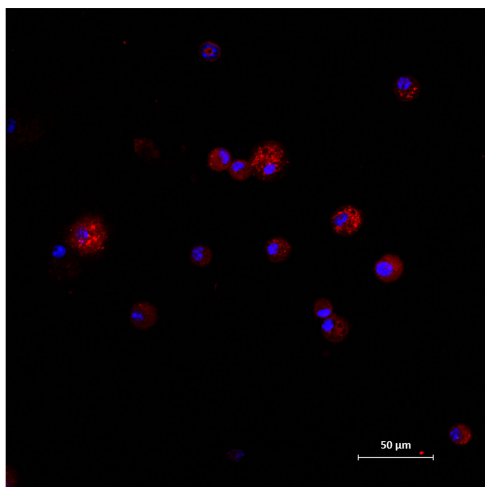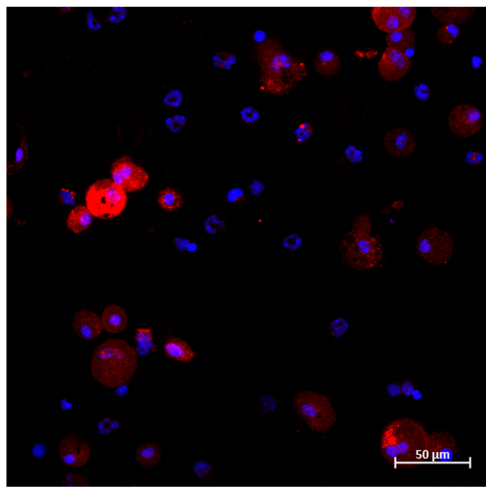

Supplement: Supplementary file 1 [file ijms-23-00047-s001.zip › supplemental figure S5.pdf]

DAPI / Ext. MFG-E8

C57BL/6J

SHAM

MWCNT

ABCG1KO

SHAM

MWCNT

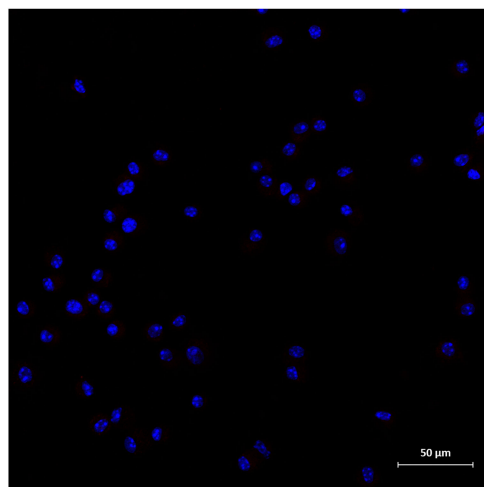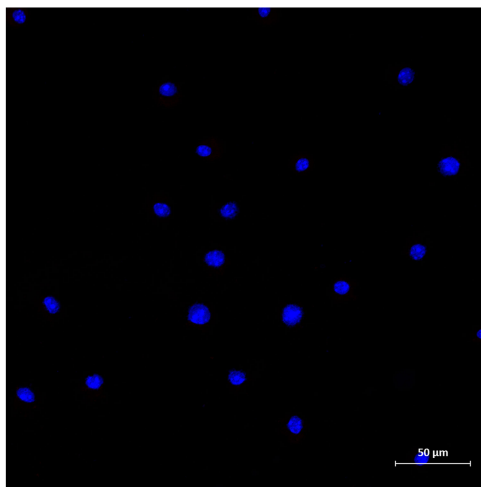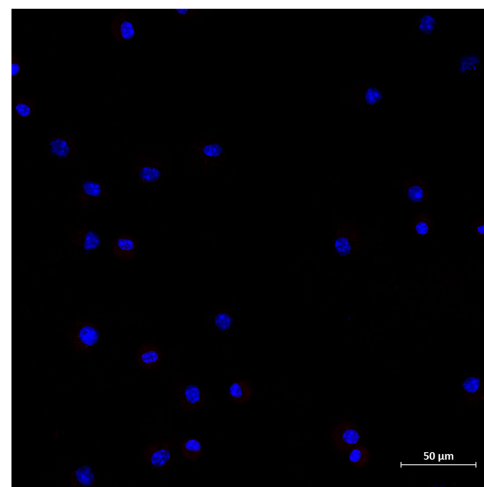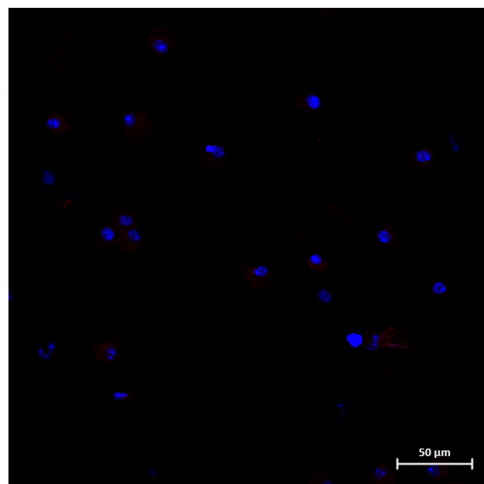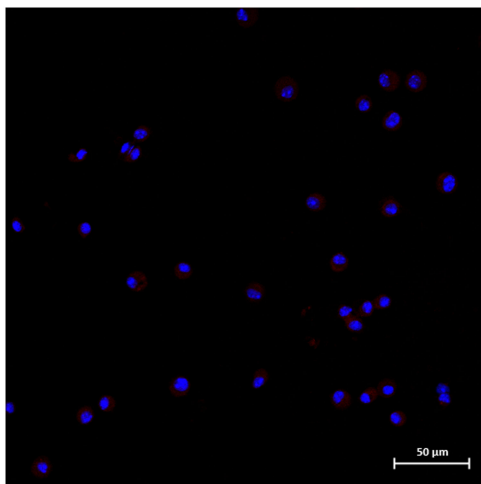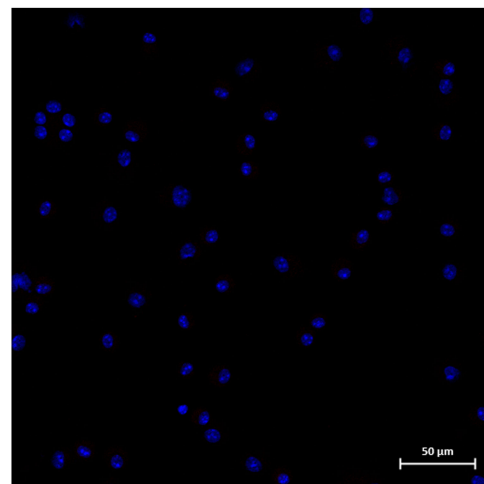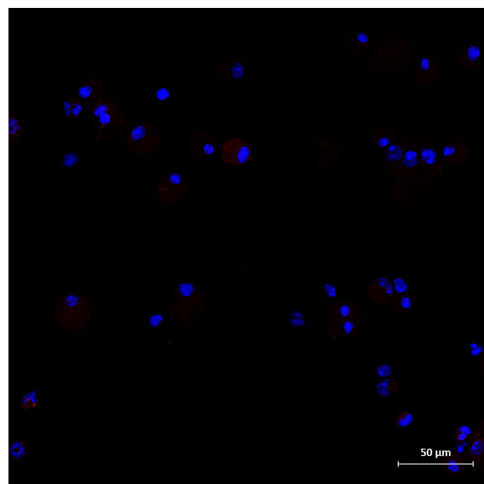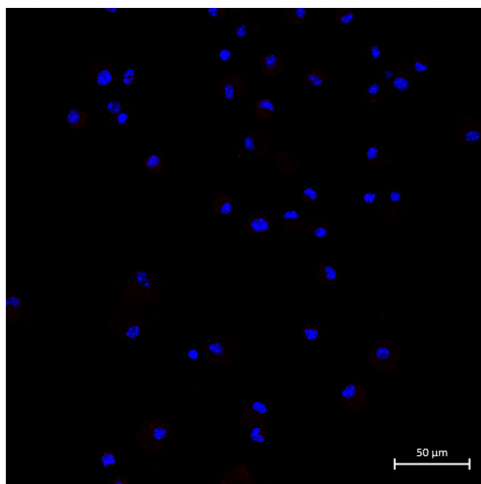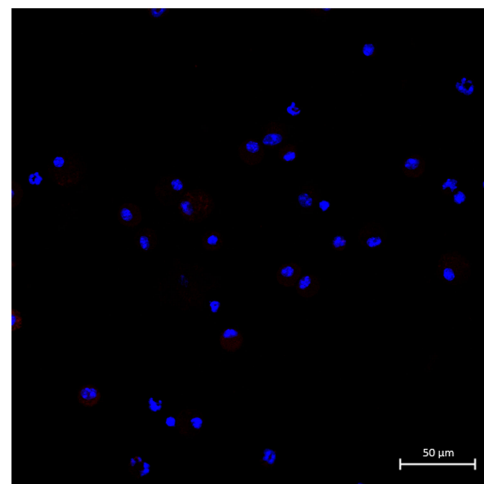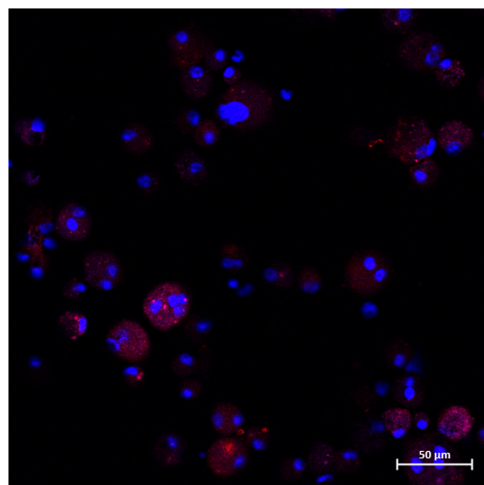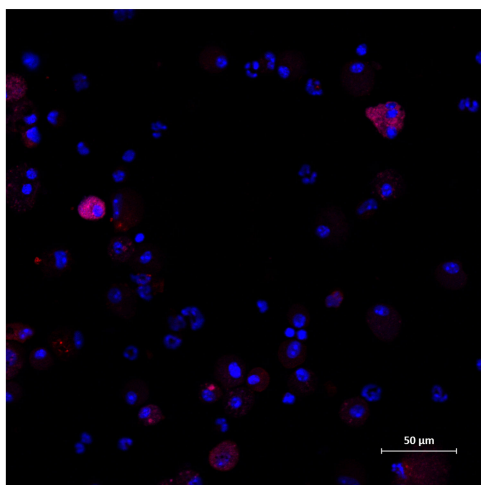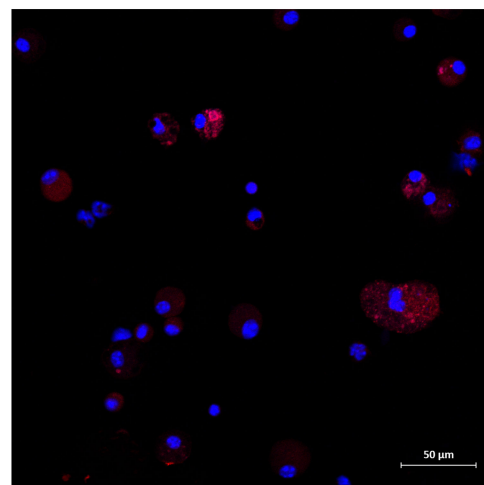

Supplement: Supplementary file 1 [file ijms-23-00047-s001.zip › supplemental figure S6.pdf]

*A*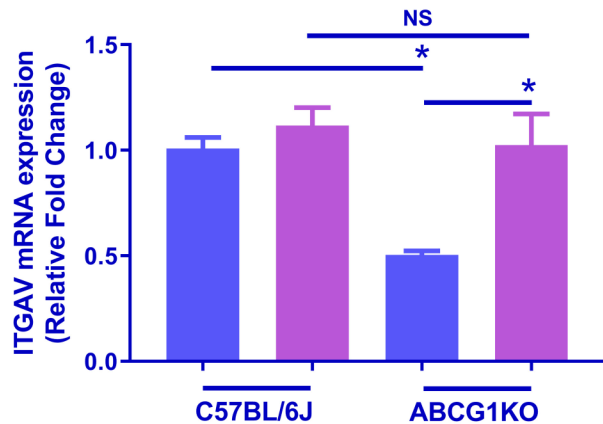*B*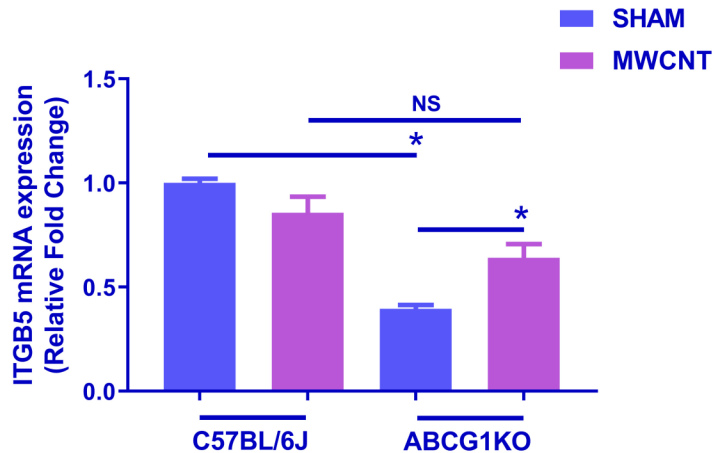

Supplement: Supplementary file 1 [file ijms-23-00047-s001.zip › supplemental figure S7.pdf]
